# The Treatment of Tuberculosis

**Published:** 1913-02-22

**Authors:** John Laird

**Affiliations:** 1 Wine Street, Sligo, February 16, 1913.


### The Treatment of Tuberculosis.

*To the Editor of* The Hospital.

Sir,—In your issue of January 25 you have been kind enough to insert a review of a little book which I have recently written—namely, "Notes on the Treatment of Tuberculosis."

My first object in writing is to correct a grave error into which the reviewer has fallen in giving the dose of tincture of pulsatilla as one to two drachms, instead of one to two minims; this should be corrected lest harm may result if anyone should wish to give the treatment a trial after reading your review.

My second object is to respectfully suggest that the reviewer has not carefully read through the different chapters, as he makes no reference to the salts of calcium which the writer considers so important and so essential in the treatment. It is the combination of the mixture, and at the same time the administration of the salts of calcium, which constitutes the full treatment.

The reviewer writes of the prescription as being of the "shot-gun" variety, yet it has taken the author over a quarter of a century to put it together in its present form!

While the reviewer commends the author for publishing the results of his work, yet is it not a pity that a journal so ably conducted should review the book after such a very superficial or partial reading of it?

I do not object to fair criticism, but rather court it. Suffice to say that the results of the treatment in my own practice have been of recent years of the most gratifying nature, and I was anxious that others should have the benefit of my years of work on the subject.

Apologising for the length of this letter,—I am yours faithfully, John Laird.

1 Wine Street, Sligo, February 16, 1913.

[Mr. Laird may be assured that the book was carefully read, and that his views upon calcium salts were not overlooked. Apologies are willingly tendered for the mistake as to the dose of pulsatilla. The number of years during which the prescription has been compiled and perfected does not alter the fact that it is of the "shot-gun" variety.—The Reviewer.]

